# ChatGPT-4o-Generated Exercise Plans for Patients with Type 2 Diabetes Mellitus—Assessment of Their Safety and Other Quality Criteria by Coaching Experts

**DOI:** 10.3390/sports13040092

**Published:** 2025-03-24

**Authors:** Samir Akrimi, Leon Schwensfeier, Peter Düking, Thorsten Kreutz, Christian Brinkmann

**Affiliations:** 1 Department of Preventive and Rehabilitative Sport Medicine, Institute of Cardiovascular Research and Sport Medicine, German Sport University Cologne, 50933 Cologne, Germany; s.akrimi@gmx.de (S.A.); lschwensfeier@gmail.com (L.S.); 2 Department of Sports Science and Movement Pedagogy, TU Braunschweig, 38106 Braunschweig, Germany; peter.dueking@tu-braunschweig.de; 3 Department of Fitness & Health, IST University of Applied Sciences, 40233 Düsseldorf, Germany; tkreutz@ist-hochschule.de

**Keywords:** exercise, training, diabetes, digital health, health benefits

## Abstract

In this discussion paper based on preliminary data, the safety and other quality criteria of ChatGPT-4o-generated exercise plans for patients with type 2 diabetes mellitus (T2DM) are evaluated. The study team created three fictional patient profiles varying in sex, age, body mass index, secondary diseases/complications, medication, self-rated physical fitness, weekly exercise routine and personal exercise preferences. Three distinct prompts were used to generate three exercise plans for each fictional patient. While Prompt 1 was very simple, Prompt 2 and Prompt 3 included more detailed requests. Prompt 3 was optimized by ChatGPT itself. Three coaching experts reviewed the exercise plans for safety and other quality criteria and discussed their evaluations. Some of the exercise plans showed serious safety issues, especially for patients with secondary diseases/complications. While most exercise plans incorporated key training principles, they showed some deficits, e.g., insufficient feasibility. The use of more detailed prompts (Prompt 2 and Prompt 3) tended to result in more elaborate exercise plans with better ratings. ChatGPT-4o-generated exercise plans may have safety issues for patients with T2DM, indicating the need to consult a professional coach for feedback before starting a training program.

## 1. Introduction

Tailored exercise interventions and physical activity are cornerstones in managing type 2 diabetes mellitus (T2DM), providing benefits such as improved insulin sensitivity and reduced cardiovascular disease risk [1,2]. Designing exercise plans for patients with T2DM presents particular challenges. Factors such as secondary complications and individual preferences, among other things, must be considered to ensure safe training and encourage adherence to the exercise program [3]. Therefore, developing exercise plans for patients with T2DM requires expert knowledge and is time-consuming. Tailored exercise plans are not available for all patients with T2DM, especially not for those lacking access to experienced professional coaches. To overcome these problems, types of artificial intelligence (AI), such as Large Language Models (LLMs) might be useful.

LLMs such as ChatGPT have the potential to address health-related issues [4]. Launched in November 2022 by OpenAI, ChatGPT is an AI-powered LLM trained on extensive datasets, allowing it to generate human-like text [5]. According to OpenAI’s technical report, GPT-4 exhibits capabilities comparable to those of human experts in certain complex professional and academic tasks [6]. However, ChatGPT has received mixed reactions within academia and the broader scientific community, reflecting an ongoing debate about the benefits and risks associated with AI technology [7]. While ChatGPT offers significant potential, it also has certain limitations, such as the generation of fictitious references and artificial hallucinations [8,9]. Moreover, Li et al. [10] identified additional limitations, including the tendency to present inaccurate information in a convincingly authoritative manner.

Customer opinion on AI in healthcare and sports is shaped by (a lack of) trust, perceived benefits and ethical considerations [11,12]. According to the Technology Acceptance Model (TAM), people are more likely to use AI technology if they perceive it as useful and easy to use [13]. In healthcare, the potential of AI to improve diagnosis and treatment is widely recognized, but concerns remain, e.g., regarding data privacy, decision-making autonomy or the erosion of the therapist–patient relationship [12]. These concerns highlight the need to increase public trust by creating a framework that balances innovation with ethical considerations [14].

Evidence on the quality of ChatGPT-generated exercise plans remains limited, which is not surprising given the novelty of research in this area. Düking et al. [15] evaluated the quality of ChatGPT-generated exercise plans for healthy athletes. Their findings showed that the quality of exercise plans generated by ChatGPT for runners was highly dependent on the information provided to the LLM, and that the best rated exercise plan was not optimal.

Due to the increasing interest in AI and ChatGPT in the healthcare sector and particular challenges in creating exercise plans for T2DM patients, this article contributes to the following research questions:

- Are exercise plans generated by ChatGPT-4o safe for patients with T2DM?
- Do ChatGPT-4o-generated exercise plans align with the American Diabetes Association (ADA) and the American College of Sports Medicine (ACSM) recommendations for physical activity in T2DM patients [1,16]?
- How do the safety and overall quality of ChatGPT-4o-generated exercise plans vary based on the quality of the prompts used?

## 2. Materials and Methods

This study was conducted in accordance with the Declaration of Helsinki. To develop and evaluate ChatGPT-generated exercise plans, we adopted the methodology used in previous studies [15,17]. Specifically, we (i) defined three fictional patients and specific prompts entered in ChatGPT-4o which differed in terms of the information provided, (ii) defined the quality criteria for the assessment of ChatGPT-4o-generated exercise plans and (iii) involved experienced raters in assessing the generated exercise plans based on the defined criteria.

### 2.1. Generating Exercise Plans Using ChatGPT-4o

The free-of-charge ChatGPT-4o software (OpenAI, L.L.C., San Francisco, CA, USA) was used to create exercise plans tailored to individuals with T2DM.

To reflect a variety of users with T2DM, the study team created three fictional patient profiles that differed in terms of sex, age, body mass index, secondary complications, medication, self-rated physical fitness, weekly exercise routines and personal exercise preferences (Table 1).

Using this information, the following prompts were used to generate exercise plans (see Table 2). Prompt 1 was very basic, while Prompt 2 was more specific. The third prompt was refined by ChatGPT itself in line with Giray’s prompt engineering guidelines [18] using Prompt 2 as the starting prompt.

Using these prompts and each patient’s specific information, a total of nine exercise plans were generated on 16 September 2024.

The ChatGPT input for prompt engineering as well as all generated exercise plans are provided in Supplementary Material S1.

### 2.2. Assessment of the Quality of ChatGPT-4o-Generated Exercise Plans

Three authors (SA, LS, CB) participated in rating the exercise plans. All three authors are experienced professional training experts with academic backgrounds in sports and therapy sciences (holding at least a bachelor’s degree in sports science) and have acquired a qualification in diabetes management during their studies. All raters are members of the “Diabetes, sports and exercise” research group at the German Sport University Cologne and have contributed to multiple exercise research projects involving T2DM patients.

Quality criteria were defined in an initial roundtable discussion between the raters based on the American Diabetes Association (ADA) and the American College of Sports Medicine (ACSM)’s recommendations for physical activity in T2DM patients [1,16]. Where appropriate, detailed indicators were defined for each quality criterion (Table 3 and Table 4, left column). Quality criteria were classified as either ‘L: low’, ‘M: moderate’, ‘S: strong’ or ‘N/A: not applicable’. A rating of ‘low’ implies that the quality criterion was not sufficiently met or that something planned could potentially be dangerous for patients, ‘moderate’ that the quality criterion was partially met and ‘high’ that it was (almost) fully met. After the criteria had been individually and independently assessed by the raters (at home), they met for a joint discussion. In the case of divergent ratings, the decision was made by a majority (2:1) or, if all three raters had arrived at different ratings, the middle rating, i.e., ‘moderate’ was assigned.

The intraclass correlation coefficient (ICC) was calculated to estimate interrater reliability using IBM SPSS Statistics for Windows, version 30 (IBM Corp., Armonk, NY, USA). The ICC was 0.763 (95% CI: 0.669, 0.834), indicating ‘good’ agreement between the raters [19].

## 3. Results

### 3.1. Safety Issues

The safety ratings for the exercise plans are presented in Table 3. Several plans exhibited considerable safety gaps:

- The most common safety concern across all exercise plans was that users were not made aware of the need for a medical check-up prior to starting the training program, especially in cases of secondary conditions necessitating such a check-up (Patients 2 and 3) or when high-intensity workouts were recommended for sedentary patients [1].

Potential contraindications were generally taken into consideration, with only a few exceptions:

- High-intensity training was recommended for Patient 3 with proliferative retinopathy. High-intensity training is not recommended for patients with proliferative retinopathy due to the risk of triggering vitreous hemorrhage or retinal detachment [1].

All training plans included additional safety guidelines. However, some information was missing, e.g.:

- It was not always mentioned that patients with (insulin-treated) diabetes should monitor their glucose levels before, during and after exercise to avoid hypoglycemia [16].
- It was not always mentioned that patients with high blood pressure should avoid holding their breath during strength exercises to prevent exorbitantly high and dangerous blood pressure peaks [16].

**Figure 1:**
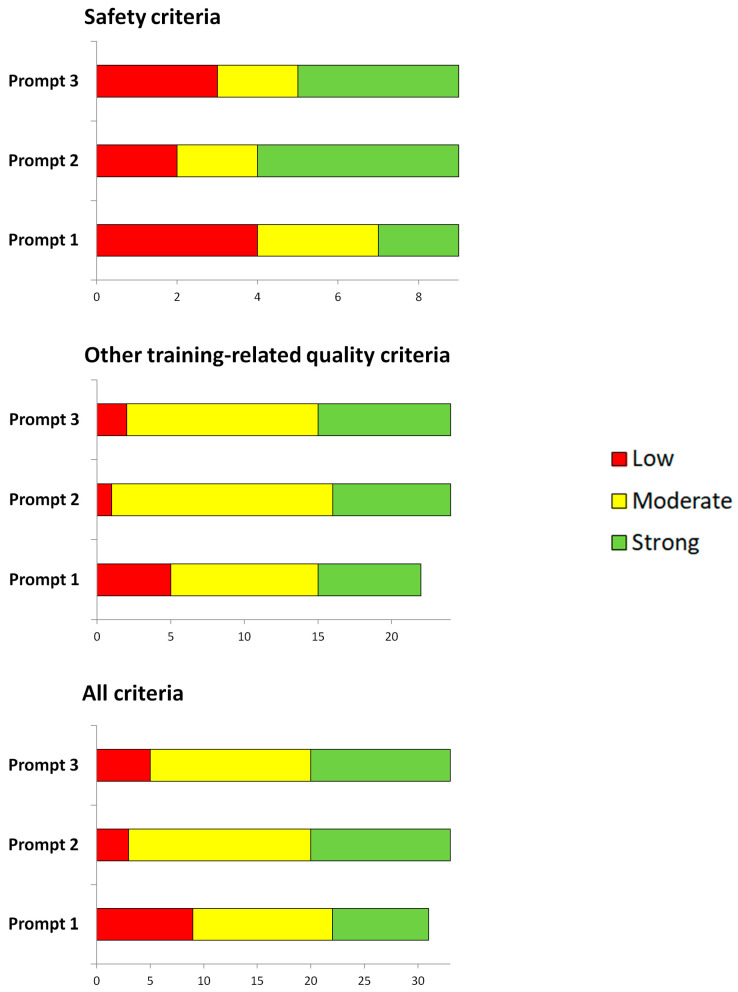
Frequency distribution of the ratings ‘low’, ‘moderate’ and ‘strong’ across all fictional patients depending on the prompts used.

Using more detailed prompts (Prompts 2 and 3) generally resulted in minimal quality improvement (Figure 1, Prompt 1: 4× L, 3× M, 2× S, Prompt 2: 2× L, 2× M, 5× S, Prompt 3: 3× L, 2× M, 4× S), primarily in terms of tailoring the plans for individuals with secondary complications by avoiding conflicts between training and potential contraindications and providing additional safety instructions.

### 3.2. Other Training-Related Quality Criteria

Training-relevant quality criteria beyond safety are presented in Table 4. Most exercise plans included a clear training goal, although this goal was not always very specific. The training content usually matched the broad goals provided, and most training plans adhered to the ADA’s recommendations for physical activity. All training plans included guidance on progressive training load increases. In many cases, the programs’ feasibility was questioned. Personal preferences were always taken into account. The most common shortcomings were:

- Training goals were not always closely aligned with SMART principles (specific, measurable, achievable, relevant, time-bound).
- Contrary to the ADA’s recommendations, no balance exercises were suggested for the older fictional patient (Patient 3).
- Missing details on the level of intensity, e.g., target training heart rate or whether to train to muscle failure during strength exercises or not.
- Many plans only included sample exercises, and it was sometimes unclear whether the user could or should add further exercises.
- The total training time did not match the proposed exercise volume.
- Many exercises seemed overwhelming, i.e., some complex bodyweight exercises seemed inappropriate for beginners.

Using more detailed prompts (Prompts 2 and 3) sometimes led to improvements (e.g., in terms of a defined training goal and/or an appropriate progressive increase in training volume and/or intensity). However, in some cases, the quality was obviously not changed or declined minimally (in terms of the feasibility of the program or its alignment with personal preferences) (Figure 1, Prompt 1: 5× L, 2× N/A, 10× M, 7× S, Prompt 2: 1× L, 15× M, 8× S, Prompt 3: 2× L, 13× M, 9× S).

## 4. Discussion

The aim of this article was to evaluate the safety and other quality criteria of ChatGPT-generated exercise plans for patients with T2DM by coaching experts.

In this exploratory study based on preliminary data, two distinct prompts (Prompt 1 and Prompt 2) were used, reflecting inputs that most users would likely enter in a similar manner. Additionally, a third prompt was refined through prompt engineering by ChatGPT to generate the highest achievable output quality.

Some exercise plans revealed significant safety concerns. There was no guidance on obtaining a medical check-up before starting the training program, where this would be appropriate. Many, but not all possible safety instructions were mentioned by ChatGPT. However, this could pose serious risks. The training plans were usually unsafe for the study’s fictional patients with diabetes, particularly for those with secondary diseases. Nearly all training plans adhered to important training principles. However, there were some deficiencies in accurately controlling training intensity and ensuring overall feasibility.

These findings are partially consistent with previous studies. Düking et al. [15] rated ChatGPT training plans for healthy runners, Washif et al. [17] for healthy strength athletes, while Dergaa et al. [20] assessed training plans for patients with various conditions (but only one of them with T2DM). All research groups found that ChatGPT-generated plans do not (yet) meet the standards of coaching experts, primarily due to limitations in complexity and the level of detail.

Individuals with T2DM should aim for at least 150 min of moderate-intensity physical activity or 75 min of vigorous-intensity physical activity per week, in addition to engaging in strength training at least twice a week. Older adults with diabetes should also consider flexibility and balance exercises [1,16]. While most exercise plans in the present study adhered to general practice guidelines, they often lacked instructions and only provided sample exercises. Guidance on how to perform exercises, especially complex resistance exercises, would be useful for patients and could help prevent potential injuries [20,21].

One positive aspect is that all exercise plans were tailored to the user’s personal preferences, which is crucial since enjoyment plays an important role in adherence to the training program [22].

On the downside, there are some serious safety risks, some of which have already been mentioned. For example, ChatGPT recommended a vigorous-intensity exercise program for patients with proliferative retinopathy. This could have serious consequences, such as triggering vitreous hemorrhage or retinal detachment [16]. In many cases, important safety instructions were absent, e.g., guidance on monitoring glucose levels over an adequate post-exercise period, which is crucial for insulin-treated patients to reduce the risk of exercise-induced hypoglycemic events [23]. Similarly, Dergaa et al. [20] noted a lack of additional safety instructions, e.g., regarding glucose monitoring or foot care. However, they described only one case scenario with a T2DM patient who was only taking metformin and did not have other diseases/any secondary complications.

The safety deficiencies observed among the different cases in the present study raise the question of the ethical responsibility of AI developers (e.g., OpenAI) in ensuring that their tools provide safe and reliable recommendations for vulnerable groups like T2DM patients. It may be an ethical obligation to inform users of the limitations and risks of relying on AI-generated plans without professional oversight, including risks of unsafe recommendations. There is a need for built-in safeguards in AI systems to reduce the risks when used in medical or fitness contexts. Ultimately, this raises questions about liability in case of adverse events. Another pressing issue is data privacy when patient data are entered and processed. Clear rules and laws are needed to protect users [24]. Patients must be informed and consent to how their data are used, ensuring full transparency [25].

In the present study, the overall quality of exercise plans improved with more detailed prompts (Prompt 2 and Prompt 3). Düking et al. [15] used different prompts and also reported an increase in overall quality when providing more inputs. It can be assumed in this context that the majority of users are more likely to use simple prompts and may not use a complex prompt or prompt engineering to optimize their queries. As a result, many users may receive an exercise plan of lower quality. On average, prompt engineering did not lead to a further improvement in quality when comparing the exercise plans for Prompt 2 and Prompt 3. This could be explained by the reason that Prompt 2 already contained many details for the query. However, this approach could be helpful, especially for inexperienced users, to achieve a better result.

Future research should investigate the potential of creating tailored models for the target group. Making a “custom GPT” (customgpt.ai, 07.01.2025) could be distributed to the target group through links or QR codes, enhancing accessibility and usability. Future AI applications could be optimized by integrating user feedback loops or collaborative AI–human decision-making systems to increase the perceived level of optimal support and improve their ability to generate safer and more tailored exercise plans [26].

A strength of the present study is the inclusion of different fictional patients and of different prompts for each of these fictional patients which differed in the information provided to ChatGPT-4o. While this allowed a thorough analysis of ChatGPT-4o’s capabilities in generating exercise plans for patients with T2DM, the results of our study might differ for other use cases and other versions of ChatGPT or other LLMs. Future research needs to critically evaluate other LLMs and use cases for LLMs. Additionally, as we showed that results differ depending on the input information provided, the users of LLMs need to be educated on how to use LLMs safely and effectively in practice.

However, some limitations remain. The study is based on only three fictional patients, limiting the generalizability of the findings. Using fictional profiles instead of real patient data simplifies the creation of different patient scenarios (with no to several secondary diseases) but does not necessarily make it possible to capture the complexity of real-world cases. Since all raters are part of the research team, inherent biases may exist. Future studies should therefore be conducted using real patient data, involving a larger and more diverse sample and external raters (blinded to the prompts used).

## 5. Conclusions

Our results imply that ChatGPT-4o-generated exercise plans are not (yet) fully safe for patients with T2DM, especially not for those with secondary diseases/complications. While some plans effectively address various training-related aspects, others clearly fall short. Using more detailed prompts generally yields more elaborate exercise plans. However, based on our analysis, the results of this study imply that exercise plans created with ChatGPT-4o for individuals with T2DM are not able to provide fully individualized guidance tailored to the specific needs of the patients. When T2DM patients use ChatGPT-4o to generate exercise plans, it is recommended to involve a professional exercise coach to provide feedback before the training. It is also conceivable that ChatGPT can assist exercise coaches in designing exercise plans. In both scenarios, there is a high potential for AI–human collaboration to enhance exercise planning outcomes. Coaches should pay particular attention to whether a prior medical check-up is advisable and whether any precautions due to secondary diseases need to be considered. In addition, the training goal should be formulated in detail, and the selection of exercises should be critically reviewed. To improve the quality of the prompts used, patients or coaches should provide as much detail as possible and request that the current guidelines of the relevant medical societies should be followed.

## Figures and Tables

**Table 1:** Details of the fictional patients.

Patients	Sex	Age [Years]	Body Mass Index [kg/m^2^]	Secondary Complication(s)	Medication	Self-Rated Fitness Level (Low, Moderate, High)	Weekly Exercise Routine	Personal Exercise Preference
Patient 1	Female	35	28	None	Metformin	Moderate	Dancing with her husband once a week for 1 h	Aerobics
Patient 2	Female	51	45	High blood pressure, grade 1 (140–159 mmHg systolic, 90–99 mmHg diastolic)	Metformin, Ramipril	Low	None	Fitness training
Patient 3	Male	65	31	Proliferative retinopathy, diabetic foot syndrome, Wagner 0 (risk foot, no injury)	Metformin, Insulin	Low	None	Cycling

**Table 2:** Prompts used for the creation of exercise plans.

Prompts	Precise Wording
Prompt 1	“Create a 12-week exercise plan tailored to the following individual with type 2 diabetes mellitus: [individual patient details, e.g., female, 35 years old, body mass index of 28 kg/m^2^, …]”
Prompt 2	“Create a 12-week exercise plan tailored to the following individual with type 2 diabetes mellitus: [individual patient details]. Consider recommendations from the American Diabetes Association and the American College of Sports Medicine. Consider potential contraindications. Define a possible training goal. Specify training type, frequency per week, duration of a single training session, training method and training intensity. If possible, also consider the individual’s personal exercise preference”.
Prompt 3	“Instruction: Create a detailed, 12-week exercise plan specifically tailored for an individual with type 2 diabetes mellitus. The exercise plan should align with established medical and fitness guidelines, while also addressing the individual’s personal preference and health status. Context: The individual is … [individual patient details]. The American Diabetes Association (ADA) and the American College of Sports Medicine (ACSM) provide guidelines for exercise plans for people with type 2 diabetes, which should be considered to ensure safety and effectiveness. Any contraindications that might arise from the individual’s medical condition or medications should be taken into account. The person prefers ….[individual patient details] and the plan should balance aerobic activities with other types of exercises beneficial for managing diabetes, such as resistance training. Input data: Using the given information, develop an exercise plan that specifies: Training goal: Define a realistic and attainable training goal for the 12-week period, considering weight management, improved insulin sensitivity, or cardiovascular health. Training type: Include both aerobic and resistance training components in the plan, while giving priority to the individual’s preference for … Frequency per week: Indicate how often the individual should exercise per week for optimal benefits. Duration per session: Specify how long each session should last. Training method and intensity: Detail the type of training methods to be used (e.g., high-intensity interval training, steady-state cardio, circuit training, etc.) and recommend appropriate intensity levels (e.g., moderate or vigorous) based on the individual’s fitness level and health condition. Considerations: Incorporate any specific exercise adjustments or precautions relevant to his/her condition, medication and personal preference (e.g., metformin side effects, possible blood sugar management during exercise). Output indicator: Provide the response in the form of a structured 12-week exercise plan. Break it down week by week and include a summary of each week’s focus. The plan should cover the following for each week: the type of exercise, the number of sessions per week, the duration of each session, the training method and intensity. Also, include a brief explanation of how the plan adheres to ADA and ACSM guidelines, and how it addresses both the individual’s health needs and his/her personal exercise preference”.

**Table 3:** Assessment of the ChatGPT-4o-generated exercise plans: safety issues.

Safety Criteria (According to ElSayed et al. [16]; Kanaley et al. [1])	Exercise Plan 1 (Patient 1, Prompt 1)	Exercise Plan 2 (Patient 2, Prompt 1)	Exercise Plan 3 (Patient 3, Prompt 1)	Exercise Plan 4 (Patient 1, Prompt 2)	Exercise Plan 5 (Patient 2, Prompt 2)	Exercise Plan 6 (Patient 3, Prompt 2)	Exercise Plan 7 (Patient 1, Prompt 3)	Exercise Plan 8 (Patient 2, Prompt 3)	Exercise Plan 9 (Patient 3, Prompt 3)
Advice for a medical check-up prior to the start of the training program (for individuals who are older than 40 years of age, have any secondary complications, intend to undertake high-intensity workouts, intend to undertake high-intensity physical activities and are currently sedentary adults or have a diabetes duration > 10 years)	L	L	L	S	L	L	L	L	L
No conflicts with possible contraindications (e.g., high-intensity training for patients with proliferative retinopathy or weight-bearing exercises for patients with diabetic foot ulcers)	S	S	L	S	S	M	S	S	S
Additional safety instructions (e.g., for patients with insulin treatment: regularly check glucose values before/during/after exercise; for all patients with diabetes: stay hydrated; for patients with hypertension: do not hold breath during strength exercises)	M	M	M	S	S	M	S	M	M

**Table 4:** Assessment of the ChatGPT-4o-generated exercise plans: training-related quality criteria.

Training-Related Quality Criteria (According to Brinkmann [3]; Düking et al. [15]; ElSayed et al. [16]; Washif et al. [17])	Exercise Plan 1 (Patient 1, Prompt 1)	Exercise Plan 2 (Patient 2, Prompt 1)	Exercise Plan 3 (Patient 3, Prompt 1)	Exercise Plan 4 (Patient 1, Prompt 2)	Exercise Plan 5 (Patient 2, Prompt 2)	Exercise Plan 6 (Patient 3, Prompt 2)	Exercise Plan 7 (Patient 1, Prompt 3)	Exercise Plan 8 (Patient 2, Prompt 3)	Exercise Plan 9 (Patient 3, Prompt 3)
Suitable specific training goal (SMART: specific, measurable, achievable, relevant, time-bound)	L	M	L	M	M	M	M	M	M
Goal-specific training content if training goal has been defined	N/A	M	N/A	M	M	M	M	M	M
Minimum volume and intensity for recommended types of exercise (endurance, strength, flexibility and balance) in alignment with ADA’s guidelines (intended target)	S	M	L	S	M	L	S	S	L
Adequate progressive loads during the training period for the proposed training program	M	M	M	M	M	S	S	S	M
Monitoring exercise loads (e.g., heart rate, subjective rating of perceived exertion)	L	M	L	M	M	S	M	M	L
Feasibility of the program	M	M	S	M	M	S	M	M	M
Considering the individual’s initial performance level/self-rated physical fitness	S	S	S	S	S	S	S	S	S
Considering personal preferences	M	S	S	M	M	S	S	M	S

## Data Availability

The original contributions presented in the study are included in the article/Supplementary Materials, further inquiries can be directed to the corresponding author.

## Supplementary Materials

The following supporting information can be downloaded at: https://www.mdpi.com/article/10.3390/sports13040092/s1, File S1: ChatGPT input for prompt engineering; ChatGPT-4o-generated exercise plans.

## Abbreviations

The following abbreviations are used in this manuscript:

ACSM	American College of Sports Medicine
ADA	American Diabetes Association
AI	Artificial intelligence
ICC	Intraclass correlation coefficient
LLM	Large Language Models
T2DM	Type 2 diabetes mellitus
TAM	Technology Acceptance Model
