# Supplementary material for: ChatGPT-4o-Generated Exercise Plans for Patients with Type 2 Diabetes Mellitus—Assessment of Their Safety and Other Quality Criteria by Coaching Experts
Source: Sports (Basel). 2025 Mar 24;13(4):92. doi: 10.3390/sports13040092 (PMC12031090; doi:10.3390/sports13040092)
Supplement: Supplementary file 1 [file sports-13-00092-s001.zip › sports-3453184-supplementary.pdf]

Supplementary Material 1

ChatGPT input for prompt engineering:

”Please consider yourself an expert in prompt engineering for ChatGPT and help me with the following prompt. Your task is to optimize it to achieve the best possible response. When optimizing, stick to the implementation of the following four elements (examples are given below), which must be included in a good prompt. Ask if you are not sure what the four elements should contain:

(1) Instruction: A specific task or instruction that guides the model’s behavior and directs it toward the desired output.

(2) Context: External information or additional context that provides background knowledge to the model, helping it generate more accurate and relevant responses.

(3) Input data: The input or question that we want the model to process and provide a response for. It forms the core of the prompt and drives the model’s understanding of the task.

(4) Output indicator: Specifies the type or format of the desired output. It helps shape the response by defining whether we need a short answer, a paragraph, or any other specific format.

Here is an example from the field of biomedical engineering that illustrates the meaning of the 4 elements:

(1) Instruction: “Write an essay discussing the role of nanotechnology in targeted drug delivery for cancer treatment.”

(2) Context: “Explore the applications of nanotechnology in biomedical engineering, focusing on its potential to improve the effectiveness and safety of cancer treatments through targeted drug delivery systems.”

(3) Input data: “Provide an overview of nanotechnology-based drug delivery systems, such as nanoparticles or nanocarriers, and their ability to selectively deliver anticancer drugs to tumor sites. Discuss the advantages, challenges, and potential future advancements in this field.”

(4) Output indicator: “Please present your findings in a well-structured essay format, including an introduction, main body paragraphs covering key aspects of nanotechnology in drug delivery, and a conclusion. Aim for approximately 1500 words.”

This is the prompt to optimize:

“Create a 12-week exercise plan tailored to the following individual with type 2 diabetes mellitus: [individual patient details].

Consider recommendations from the American Diabetes Association and the American College of Sports Medicine. Consider potential contraindications. Define a possible training goal. Specify training type, frequency per week, duration of a single training session, training method and training intensity. If possible, also consider the individual’s personal exercise preference.”

Now please give me the optimized prompt.“

ChatGPT-4o-generated exercise plans:

Patient 1, Prompt 1

Here’s a 12-week aerobics-focused exercise plan tailored to your preferences and fitness level, integrating your weekly dancing routine and aiming to improve your overall fitness and manage your type 2 diabetes.

Weeks 1-4: Foundation Phase

Focus on building endurance, establishing routine, and preventing injury.

Week 1 & 2:

- **Aerobics (Low-Impact)**: 3 times a week, 30 minutes/session
  - Example: Low-impact cardio routines (marching in place, side steps, step touches)
- **Strength Training**: 2 times a week, 20 minutes/session
  - Focus on bodyweight exercises (squats, lunges, push-ups, planks)
- **Dancing**: 1 hour of your weekly dancing routine
- **Stretching**: 10 minutes after each session

Week 3 & 4:

- **Aerobics (Moderate-Impact)**: 3 times a week, 35 minutes/session
  - Increase intensity with more dynamic moves (knee lifts, light jumping jacks)
- **Strength Training**: 2 times a week, 25 minutes/session
- **Dancing**: Continue with your 1-hour weekly session
- **Stretching & Balance Work**: 10-15 minutes after each session

Weeks 5-8: Progression Phase

Increase intensity and introduce more complex movements.

Week 5 & 6:

- **Aerobics (Moderate-Impact)**: 3 times a week, 40 minutes/session
  - Example: Kickboxing, dance aerobics, or step aerobics
- **Strength Training**: 2 times a week, 30 minutes/session
  - Add light dumbbells (1-3 kg) for squats, lunges, and arm exercises
- **Dancing**: Keep your 1-hour weekly dance

- **Core Work**: Add 5-10 minutes of core-focused exercises (planks, Russian twists)

Week 7 & 8:

- **Aerobics (High-Impact Mix)**: 3 times a week, 45 minutes/session
  - Alternate between high-intensity intervals and moderate pace
- **Strength Training**: 2 times a week, 35 minutes/session
  - Increase weights if comfortable
- **Dancing**: Continue with your 1-hour weekly session
- **Stretching & Core Work**: 10-15 minutes

Weeks 9-12: Peak Phase

Push limits safely and enhance aerobic capacity.

Week 9 & 10:

- **Aerobics (High-Impact)**: 4 times a week, 45-50 minutes/session
  - Include a mix of high-intensity intervals, dance, and step aerobics
- **Strength Training**: 2 times a week, 40 minutes/session
  - Incorporate more challenging exercises with weights or resistance bands
- **Dancing**: 1-hour weekly session
- **Stretching & Core Work**: 15 minutes post-workout

Week 11 & 12:

- **Aerobics (High-Intensity Intervals)**: 4 times a week, 50 minutes/session
  - Alternate 1 minute of high-intensity (jump squats, burpees) with 2 minutes of moderate intensity
- **Strength Training**: 2 times a week, 45 minutes/session
- **Dancing**: Continue with your weekly 1-hour session
- **Cooldown & Flexibility**: 15-20 minutes after each workout

Notes:

- **Blood Sugar Monitoring**: Check blood sugar levels before and after exercise, especially with more intense workouts.
- **Hydration**: Drink plenty of water, especially before and after aerobic sessions.
- **Rest**: Ensure 1-2 rest days per week to avoid overtraining.

Let me know if you'd like to adjust anything!

Patient 2, Prompt 1

Creating a safe and effective 12-week exercise plan for a 51-year-old female with type 2 diabetes mellitus, a BMI of 45, high blood pressure (grade 1), and a low fitness level will prioritize gradual progress and monitoring of health. The focus will be on improving cardiovascular health, managing blood sugar, reducing blood pressure, and promoting weight loss. Here's a suitable plan with fitness training as the core focus:

Guidelines:

- **Frequency:** Begin with 3 sessions per week, progressing to 5 sessions in later weeks.
- **Duration:** Start with short 20–30-minute sessions, gradually increasing to 45 minutes.
- **Intensity:** Begin with low intensity, progressing to moderate intensity.
- **Monitoring:** Check blood pressure before and after workouts and monitor blood glucose levels regularly.
- **Rest:** Ensure at least one rest day between workout days in the early weeks.

Weeks 1-4: Foundation Phase (Building Baseline Fitness)

Goals: Increase movement, improve circulation, and build cardiovascular endurance.

- **Frequency:** 3 days/week (e.g., Monday, Wednesday, Friday)
- **Duration:** 20–30 minutes/session
- **Intensity:** Low (RPE 3–4 on a 10-point scale)

Exercises:

- **Warm-up (5 minutes):** Slow walk or stationary cycling at an easy pace.

Workout:

1. **Cardio (10–15 minutes):**
    - Walking (on a treadmill or outdoors) or recumbent cycling. Start slow and focus on steady movement, aiming for a pace that allows conversation.
2. **Bodyweight Strength Exercises (2 sets of 8–10 reps each):**
    - Wall Push-ups
    - Sit-to-stand (chair squats)
    - Seated Leg Extensions (use a chair for stability)
    - Seated Knee Raises (to engage core)

Cool-down (5 minutes): Gentle stretches, focusing on legs, arms, and back.

Notes:

- Gradually increase walking pace or cycling resistance each week.

- Monitor heart rate and keep it within a safe range (consult with a healthcare provider for personalized heart rate targets).

Weeks 5-8: Progression Phase (Increasing Endurance and Strength)

Goals: Increase stamina and introduce moderate strength training.

- **Frequency:** 4 days/week (e.g., Monday, Tuesday, Thursday, Saturday)
- **Duration:** 30–35 minutes/session
- **Intensity:** Low to moderate (RPE 4–5)

Exercises:

- **Warm-up (5–7 minutes):** Brisk walking or cycling, increasing pace slightly.

Workout:

1. **Cardio (15–20 minutes):**
    - Brisk walking or using a low-impact cardio machine (e.g., elliptical or stationary bike). Try to walk a bit faster than in previous weeks.
2. **Strength Training (2–3 sets of 10–12 reps each):**
    - Modified Wall Push-ups or Incline Push-ups
    - Step-ups (on a low step or curb)
    - Seated Leg Curls (use resistance bands if comfortable)
    - Seated Dumbbell Press (light weights)

Cool-down (5 minutes): Stretching, focusing on flexibility in the major muscle groups.

Notes:

- Gradually increase resistance during strength training exercises.
- Introduce light hand weights (1-2 kg) for upper body exercises if comfortable.

Weeks 9-12: Endurance and Strength Enhancement Phase

Goals: Improve overall cardiovascular fitness and muscular strength.

- **Frequency:** 5 days/week (e.g., Monday, Tuesday, Thursday, Friday, Sunday)
- **Duration:** 35–45 minutes/session
- **Intensity:** Moderate (RPE 5–6)

Exercises:

- **Warm-up (5–7 minutes):** Moderate walking or light jogging.

Workout:

1. **Cardio (20–25 minutes):**
    - Brisk walking, elliptical, or cycling at a steady, moderate pace. Aim for an intensity where talking is possible but more difficult.
2. **Strength Training (3 sets of 12–15 reps each):**
    - Incline Push-ups
    - Step-ups (increase the height slightly)
    - Seated Dumbbell Shoulder Press
    - Seated Leg Lifts
    - Dumbbell Rows

Cool-down (5 minutes): Stretching, focusing on improving flexibility and relaxation.

Notes:

- Encourage using slightly heavier weights for strength exercises if comfortable.
- Add in light aerobic intervals (e.g., 1 minute faster walking or light jogging) to build stamina.

Additional Considerations:

- **Blood Sugar Monitoring:** Check blood glucose before and after exercise, especially during higher-intensity sessions.
- **Hydration and Nutrition:** Stay hydrated and eat a balanced meal or snack before and after exercising to manage blood sugar.
- **Gradual Progression:** Avoid sudden increases in intensity. Monitor how the body responds and adjust based on energy levels, blood pressure, and glucose control.
- **Consultation:** Regularly consult a healthcare provider to ensure safety, especially with blood pressure medications and metformin.

This plan emphasizes slow, consistent improvement while considering diabetes, hypertension, and low fitness levels. It’s important to listen to the body and adjust as necessary.

Patient 3, Prompt 1

Here's a tailored 12-week exercise plan for a 65-year-old male with type 2 diabetes mellitus, considering his complications (proliferative retinopathy, diabetic foot syndrome), low fitness level, and cycling as his preferred exercise:

General Guidelines

- **Start slowly**: Given the low fitness level, the goal is to gradually increase the duration and intensity of the sessions.

- **Foot care**: Ensure appropriate cycling footwear to minimize pressure and avoid foot injuries. Regular foot checks are essential.
- **Retinopathy consideration**: Avoid intense exercises that involve straining or bending, as these may increase the risk of retinal detachment.

12-Week Cycling Plan

Week 1-2: Building a Habit

- **Objective**: Start slowly, focusing on forming a routine.
- **Frequency**: 2 times per week.
- **Duration**: 10-15 minutes of gentle cycling per session.
- **Intensity**: Low, maintain a pace that allows comfortable conversation.
- **Notes**: Focus on smooth cycling without straining; avoid bumpy or uneven terrain.

Week 3-4: Gradual Increase

- **Objective**: Increase endurance.
- **Frequency**: 2-3 times per week.
- **Duration**: 15-20 minutes of cycling per session.
- **Intensity**: Still low, but slightly increase pace if comfortable.
- **Notes**: Consider short, flat routes with no sudden changes in elevation.

Week 5-6: Developing Consistency

- **Objective**: Build a consistent routine.
- **Frequency**: 3 times per week.
- **Duration**: 20-25 minutes per session.
- **Intensity**: Moderate; aim for a steady, moderate pace.
- **Notes**: Ensure frequent hydration and take short breaks if needed. Check feet regularly after each session.

Week 7-8: Adding Challenge

- **Objective**: Continue building endurance and cardiovascular health.
- **Frequency**: 3 times per week.
- **Duration**: 25-30 minutes per session.
- **Intensity**: Moderate, but allow brief spurts of slightly higher intensity (1-2 minutes).
- **Notes**: Keep routes flat and smooth. Monitor blood sugar levels before and after cycling.

Week 9-10: Focus on Endurance

- **Objective**: Improve cardiovascular fitness.
- **Frequency**: 3-4 times per week.
- **Duration**: 30-35 minutes per session.
- **Intensity**: Moderate with some brief periods of higher intensity cycling.
- **Notes**: Avoid overexertion. Always prioritize safety, especially foot care.

Week 11-12: Reaching Consistency and Sustainability

- **Objective**: Build a sustainable routine.
- **Frequency**: 4 times per week.
- **Duration**: 35-40 minutes per session.
- **Intensity**: Moderate with some comfortable pushes (30-60 seconds) as tolerated.
- **Notes**: Focus on maintaining this routine moving forward for long-term health benefits.

Safety and Monitoring

- **Warm-up and cool-down**: Always start and end each session with 5 minutes of gentle cycling or stretching.
- **Monitor blood sugar**: Check blood glucose levels before and after exercise, especially if on insulin.
- **Foot checks**: Given the diabetic foot syndrome, inspect feet regularly to avoid sores or blisters.
- **Eye health**: Be mindful of any visual changes and consult a healthcare provider if any occur during the program.

This gradual plan focuses on improving fitness safely while considering diabetes-related complications.

Patient 1, Prompt 2

12-Week Exercise Plan for a Female with Type 2 Diabetes Mellitus

Profile Recap:

- **Age**: 35
- **BMI**: 28
- **Condition**: Type 2 diabetes mellitus (no complications), taking metformin
- **Fitness level**: Moderate
- **Exercise preference**: Aerobics and dancing
- **Current exercise routine**: Dancing once a week (1 hour)

- **Goal**: Improve cardiovascular fitness, enhance insulin sensitivity, reduce BMI, and support weight loss

Training Goal:

- **Primary Goal**: Improve cardiovascular health and insulin sensitivity
- **Secondary Goal**: Promote weight loss by reducing BMI and increasing lean muscle mass

Weekly Plan Overview:

- **Type of Training**: Aerobic and strength training
- **Frequency**: 4-5 days/week
- **Duration**: 45-60 minutes per session
- **Training Intensity**: Moderate to moderately high (60-75% of max heart rate)
- **Methods**: Circuit training (for strength) and steady-state aerobics

Phase Breakdown

Each phase progresses in intensity and complexity to match the user's improving fitness level.

Phase 1 (Weeks 1-4)

Focus: Build endurance and consistency

- **Frequency**: 4 days/week
- **Duration**: 45 minutes per session
- **Type of Training**:
    - 3 days of aerobic exercises
    - 1 day of strength training (body weight focused)
    - 1 day of dancing (continue current routine)

Sample Aerobic Session (45 mins):

- **Warm-up**: 5-7 mins of light aerobics (marching in place, light jogging, or walking)
- **Main Workout**:
    - 30 mins of low-impact aerobics (step aerobics, cycling, brisk walking) at a moderate intensity (60-65% of max heart rate)
- **Cool-down**: 5-10 mins of stretching, focusing on major muscle groups

Sample Strength Session (45 mins):

- **Warm-up**: 5 minutes of light cardio
- **Main Workout** (Bodyweight Circuit, 2-3 rounds):

    - Squats: 12-15 reps
    - Push-ups (modified if necessary): 10-12 reps
    - Glute bridges: 12-15 reps
    - Planks: 20-30 seconds
    - Chair dips: 10-12 reps
- **Cool-down**: 5-7 minutes of stretching

Phase 2 (Weeks 5-8)

Focus: Increase intensity and introduce variety

- **Frequency**: 4-5 days/week
- **Duration**: 50 minutes per session
- **Type of Training**:
    - 3 days of aerobic exercises
    - 2 days of strength training
    - 1 day of dancing

Sample Aerobic Session (50 mins):

- **Warm-up**: 5-7 mins of dynamic stretching or light cardio
- **Main Workout**:
    - 35 mins of moderate-intensity aerobics (can include higher-intensity intervals of 1-2 minutes every 10 minutes)
    - Activities: Step aerobics, elliptical machine, or Zumba
- **Cool-down**: 5-10 mins of stretching

Sample Strength Session (50 mins):

- **Warm-up**: 5 mins of light cardio
- **Main Workout** (Strength Circuit, 3 rounds):
    - Dumbbell squats: 12-15 reps
    - Resistance band rows: 12-15 reps
    - Step-ups with bodyweight or light dumbbells: 12-15 reps
    - Wall sit: 30 seconds
    - Bicycle crunches: 12-15 reps

- **Cool-down**: 5-7 mins of stretching

Phase 3 (Weeks 9-12)

Focus: Improve stamina and strength

- **Frequency**: 5 days/week
- **Duration**: 55-60 minutes per session
- **Type of Training**:
  - 4 days of aerobic/interval training
  - 2 days of strength training
  - 1 day of dancing

Sample Aerobic Session (60 mins):

- **Warm-up**: 5-7 mins of light jogging or fast walking
- **Main Workout**:
  - 40 mins of interval training (e.g., alternating 2-3 minutes of higher intensity (70-75% max heart rate) with 2 minutes of moderate intensity)
  - Activities: Treadmill, aerobic dance classes, or cycling
- **Cool-down**: 7-10 mins of static stretching

Sample Strength Session (60 mins):

- **Warm-up**: 5 mins of light cardio
- **Main Workout** (Strength Circuit, 3 rounds with increased weights):
  - Deadlifts with dumbbells or kettlebells: 12 reps
  - Overhead press: 12 reps
  - Lunges with weights: 12 reps per leg
  - Russian twists: 15 reps per side
  - Side plank (hold for 30 seconds each side)
- **Cool-down**: 7-10 mins of stretching

General Recommendations and Considerations:

- **Monitor blood glucose levels**: Check blood sugar before and after exercise to avoid hypoglycemia, especially on metformin.
- **Stay hydrated**: Encourage adequate water intake, particularly during exercise.

- **Foot care**: Wear appropriate footwear to avoid injury, especially during weight-bearing exercises and dancing.
- **Intensity**: Exercise intensity can be monitored using the **talk test** (moderate intensity allows for conversation but not singing) or heart rate monitoring (60-75% of max heart rate).
- **Progression**: Gradually increase intensity, duration, or frequency based on the user's ability and comfort.

By the end of the 12-week program, the user should experience improvements in cardiovascular health, insulin sensitivity, and overall physical conditioning, while adhering to her preferences for aerobics and dancing.

Patient 2, Prompt 2

Here’s a 12-week exercise plan tailored to a 51-year-old female with type 2 diabetes mellitus, a BMI of 45, high blood pressure (grade 1), and a low fitness level. Considering her use of metformin and ramipril, and her personal exercise preference for fitness training, the plan emphasizes safe, progressive, and diabetes-friendly exercises. It follows recommendations from the **American Diabetes Association** (ADA) and **American College of Sports Medicine** (ACSM), and considers potential contraindications such as high blood pressure.

Training Goal:

- **Main Goal**: Improve cardiovascular health, reduce BMI, and manage blood glucose levels.
- **Secondary Goal**: Build endurance, strength, and flexibility, while controlling blood pressure.

Training Overview:

- **Type**: Aerobic exercises (walking, cycling, low-impact aerobic classes), resistance training (bodyweight, resistance bands), flexibility, and balance training.
- **Frequency**: 3–5 times per week.
- **Duration per session**: 20–45 minutes.
- **Method**: Gradual increase in exercise duration and intensity over 12 weeks.
- **Intensity**: Moderate intensity (40-60% of heart rate reserve, RPE of 3–4 on a scale of 1–10).

12-Week Exercise Plan Breakdown:

Weeks 1–4: Foundation and Cardiovascular Fitness

- **Frequency**: 3 days per week
- **Duration**: Start with 20–25 minutes per session
- **Exercise Type**:
    - **Warm-up**: 5 minutes of light walking or marching in place.
    - **Aerobic**: Walking at a comfortable pace or stationary cycling (10–15 minutes). Emphasis on low-impact activities to protect joints.

    - **Strength**: 10 minutes of basic bodyweight exercises (e.g., chair squats, wall push-ups). Start with 1–2 sets of 8–10 repetitions for each exercise.
    - **Cool-down**: 5 minutes of slow walking and gentle stretches.
- **Intensity**: Aim for moderate intensity (RPE 3–4). The user should be able to talk but not sing during exercise.
- **Notes**: Monitor blood glucose before and after exercise. Keep an eye on blood pressure response—avoid straining during resistance exercises.

Weeks 5–8: Increasing Duration and Resistance

- **Frequency**: 4 days per week
- **Duration**: 30–35 minutes per session
- **Exercise Type**:
    - **Warm-up**: 5–7 minutes of dynamic stretching or light aerobic activity (marching in place, arm circles).
    - **Aerobic**: Increase walking or cycling duration to 20 minutes. Consider adding low-impact aerobic classes or light water aerobics for variety.
    - **Strength**: Incorporate resistance bands for exercises like seated rows, leg presses (using a resistance band), and bicep curls. Perform 2–3 sets of 8–12 repetitions.
    - **Flexibility and Balance**: 5 minutes of gentle stretching (hamstrings, calves) and balance exercises (e.g., standing on one leg with support).
    - **Cool-down**: 5 minutes of slow walking or stationary cycling followed by static stretching.
- **Intensity**: Moderate intensity, maintain RPE 3–4.
- **Notes**: Gradually increase weight/resistance as strength improves. Pay attention to joint pain or blood pressure spikes.

Weeks 9–12: Building Endurance and Strength

- **Frequency**: 5 days per week
- **Duration**: 40–45 minutes per session
- **Exercise Type**:
    - **Warm-up**: 7 minutes of dynamic movements and light aerobic activity.
    - **Aerobic**: 25–30 minutes of moderate-paced walking, stationary cycling, or low-impact aerobics. If tolerated, introduce interval walking (1–2 minutes of brisk walking followed by 2 minutes of slower pace).
    - **Strength**: Continue resistance exercises with bodyweight or resistance bands. Add 1–2 new exercises like side leg raises or resistance band chest presses. Perform 2–3 sets of 10–15 repetitions.

    - **Flexibility and Balance**: Incorporate more stretching (10 minutes), focusing on improving range of motion.
    - **Cool-down**: 5 minutes of gentle stretching and slow walking or cycling.
- **Intensity**: Moderate intensity with occasional increases (RPE 4–5), especially during aerobic intervals.
- **Notes**: Regularly monitor blood glucose and blood pressure. Adjust intensity if any dizziness, shortness of breath, or joint discomfort occurs.

Training Considerations:

- **Contraindications**: Avoid exercises that increase intra-abdominal pressure (e.g., heavy lifting) as these may elevate blood pressure. Ensure proper form to prevent injury.
- **Monitoring**: Regularly check blood pressure and blood glucose before and after exercise. If glucose is below 100 mg/dL, consume a small snack before starting.
- **Progression**: Gradually increase exercise intensity and duration, allowing the body to adapt without overexertion.
- **Rest**: Ensure at least 1 rest day between strength training sessions for muscle recovery.

Lifestyle and Nutritional Tips:

- Incorporate dietary changes with guidance from a healthcare provider or dietitian to support weight loss goals and manage blood sugar levels.
- Encourage activities of daily living that promote movement, such as walking instead of driving short distances.

By the end of 12 weeks, the participant should experience improvements in cardiovascular fitness, strength, and flexibility, along with better glucose control and a reduction in blood pressure symptoms.

Patient 3, Prompt 2

Here's a 12-week cycling-based exercise plan tailored to a 65-year-old male with type 2 diabetes mellitus (BMI 31), proliferative retinopathy, diabetic foot syndrome (Wagner 0), and low fitness level. The plan is structured in line with recommendations from the **American Diabetes Association (ADA)** and the **American College of Sports Medicine (ACSM)**, focusing on gradual progression and safety.

Training Goal:

- **Improve cardiovascular fitness** while enhancing **blood sugar control**, improving **insulin sensitivity**, and **promoting weight loss**.
- **Prevent diabetic complications** from worsening by improving circulation, particularly in the lower limbs.

Considerations for Contraindications:

- **Proliferative Retinopathy**: Avoid high-impact activities or exercises that cause excessive straining (e.g., heavy lifting or intense interval training), as these may increase intraocular pressure, risking retinal detachment.

- **Diabetic Foot Syndrome (Wagner 0)**: Cycling is a low-impact option that avoids excess pressure on the feet, reducing the risk of foot injury or ulceration.

Training Approach:

- **Training Type**: Low-impact aerobic exercise (cycling).
- **Frequency**: 3-4 days per week.
- **Duration per Session**: 20-30 minutes in the beginning, progressing to 40-60 minutes over 12 weeks.
- **Training Method**: Steady-state aerobic cycling with light resistance.
- **Training Intensity**: Start with **light intensity** (40-50% of maximum heart rate), gradually increasing to **moderate intensity** (50-70% of maximum heart rate) as fitness improves.

Warm-up and Cool-down:

- **Warm-up**: 5-10 minutes of gentle cycling at a slow pace to gradually increase heart rate.
- **Cool-down**: 5-10 minutes of slower cycling to allow the heart rate to return to normal.

Week-by-Week Plan:

Weeks 1-4 (Adaptation Phase)

- **Goal**: Develop a routine, establish movement patterns, and improve circulation without overexertion.
- **Frequency**: 3 days/week.
- **Duration**: 20-25 minutes/session.
- **Intensity**: Light intensity (40-50% MHR, Rate of Perceived Exertion (RPE) 2-3 out of 10).
- **Method**: Steady-state cycling on a flat surface or low resistance on a stationary bike.
- **Rest**: At least one rest day between sessions.

Weeks 5-8 (Progression Phase)

- **Goal**: Increase cardiovascular endurance and improve blood sugar regulation.
- **Frequency**: 3-4 days/week.
- **Duration**: 25-35 minutes/session.
- **Intensity**: Progress to moderate intensity (50-60% MHR, RPE 3-4 out of 10).
- **Method**: Increase resistance slightly (or introduce mild inclines if outdoors). Begin introducing short intervals of slightly higher intensity (30-second bursts).
- **Rest**: At least one rest day between sessions.

Weeks 9-12 (Endurance Building Phase)

- **Goal**: Achieve sustained endurance and consistent blood sugar control.
- **Frequency**: 4 days/week.
- **Duration**: 35-45 minutes/session.
- **Intensity**: Moderate intensity (50-70% MHR, RPE 4-5 out of 10).
- **Method**: Continue steady-state cycling with moderate resistance. Gradually increase intensity during short intervals (45 seconds to 1 minute). Optionally, introduce gentle hills or resistance for a challenge.
- **Rest**: Active rest days with light walking or stretching to aid recovery.

Additional Recommendations:

1. **Foot Care**: Inspect feet before and after exercise to prevent foot ulcers. Wear proper cycling shoes that do not rub or cause irritation.
2. **Blood Glucose Monitoring**: Check blood glucose levels before and after exercise, especially since insulin and exercise can lower blood sugar. Keep fast-acting carbohydrates (e.g., glucose tablets) on hand.
3. **Hydration**: Stay hydrated throughout sessions, especially with potential for low blood sugar.
4. **Eyesight Protection**: Avoid rapid head movements that may increase intraocular pressure due to proliferative retinopathy.

This plan balances safety, gradual progression, and a focus on personal exercise preference (cycling), aiming to enhance overall health and manage diabetes while considering the individual's specific medical conditions.

Patient 1, Prompt 3

"12-Week Exercise Plan for a 35-Year-Old Female with Type 2 Diabetes

Overview:
The following 12-week exercise plan is designed to improve cardiovascular health, promote weight management, and enhance insulin sensitivity, in accordance with ADA and ACSM guidelines. The plan prioritizes aerobic exercises, particularly aerobics, as per the individual's preferences, while also incorporating resistance training to enhance muscle mass and improve glucose control. Training intensity is moderate, gradually progressing based on fitness improvements. Special attention is given to managing blood glucose levels during and after exercise, considering the use of metformin.

Training Goals for the 12-Week Period

- **Improve cardiovascular health** by increasing aerobic fitness.
- **Enhance insulin sensitivity** and glycemic control through regular physical activity.
- **Promote weight management** and aim for a reduction in BMI by 1–2 units, if weight loss is desired.

- **Increase muscle strength and endurance** via resistance training, which also supports glucose metabolism.

Weeks 1–4: Building Baseline Fitness and Exercise Habit

Week 1: Focus on Aerobic Conditioning and Flexibility

- **Training Frequency:** 4 days/week
- **Duration per Session:** 30–45 minutes
- **Training Type:**
    - **Aerobics (2 sessions):** Low-impact aerobics for 30 minutes/session, focusing on basic moves at moderate intensity (RPE: 5–6/10). Include 5-minute warm-up and cool-down.
    - **Dancing (1 session):** Maintain current 1-hour weekly dancing routine.
    - **Flexibility (1 session):** 30 minutes of light stretching or yoga to improve joint mobility and relaxation.
- **Training Method:** Steady-state cardio (moderate pace).
- **Intensity:** Moderate (50-60% max HR).
- **Considerations:** Monitor blood sugar before and after workouts. Hydrate regularly.

Week 2: Introduce Resistance Training

- **Training Frequency:** 4 days/week
- **Duration per Session:** 45 minutes
- **Training Type:**
    - **Aerobics (2 sessions):** Continue low-impact aerobics for 30 minutes/session.
    - **Resistance Training (1 session):** Full-body resistance training using body weight exercises (e.g., squats, lunges, push-ups). Focus on 2 sets of 10-12 reps per exercise.
    - **Dancing (1 session):** Continue with dancing for 1 hour.
- **Training Method:** Circuit training (aerobic + bodyweight resistance).
- **Intensity:** Moderate (50-65% max HR during aerobics).
- **Considerations:** Watch for signs of hypoglycemia, especially after resistance training.

Week 3: Increase Aerobic Duration

- **Training Frequency:** 5 days/week
- **Duration per Session:** 45 minutes
- **Training Type:**

    - **Aerobics (3 sessions):** Increase to 40 minutes/session, with slightly more complex movements at moderate intensity.
    - **Resistance Training (1 session):** Continue body weight exercises, add light dumbbells for additional resistance.
    - **Dancing (1 session):** Continue with dancing.
- **Training Method:** Steady-state aerobics + resistance.
- **Intensity:** Moderate (55-65% max HR).
- **Considerations:** Monitor energy levels and ensure carbohydrate intake is adequate before prolonged sessions.

Week 4: Introduce Interval Training

- **Training Frequency:** 5 days/week
- **Duration per Session:** 45 minutes
- **Training Type:**
    - **Aerobics (2 sessions):** Add intervals of higher intensity (30 seconds moderate, 30 seconds brisk).
    - **Resistance Training (1 session):** Continue with dumbbells and body weight exercises, adding 1 additional set.
    - **Dancing (1 session):** Continue dancing.
    - **Yoga or Stretching (1 session):** 30 minutes of flexibility exercises to reduce tension and increase mobility.
- **Training Method:** Interval training (HIIT) for aerobics.
- **Intensity:** Moderate to moderately high (50-70% max HR for intervals).
- **Considerations:** Ensure proper warm-up before HIIT sessions and monitor blood glucose after high-intensity intervals.

Weeks 5–8: Progression and Strength Development

Week 5: Strengthening Muscles and Endurance

- **Training Frequency:** 5 days/week
- **Duration per Session:** 50–60 minutes
- **Training Type:**
    - **Aerobics (2 sessions):** Moderate-intensity aerobics for 40 minutes, incorporating more complex choreography.
    - **Resistance Training (2 sessions):** Focus on resistance bands and free weights (3 sets of 10-12 reps).

    - **Dancing (1 session):** Maintain weekly dancing.
- **Training Method:** Circuit training with a combination of aerobics and strength exercises.
- **Intensity:** Moderate to moderately high (60-70% max HR).
- **Considerations:** Keep track of glucose levels before and after sessions to prevent hypoglycemia.

Week 6: Increase Aerobic Intensity

- **Training Frequency:** 5 days/week
- **Duration per Session:** 50–60 minutes
- **Training Type:**
    - **Aerobics (3 sessions):** Focus on interval training (HIIT) for 30 minutes followed by 10 minutes of moderate steady-state exercise.
    - **Resistance Training (1 session):** Continue using weights, increasing resistance as tolerated.
    - **Dancing (1 session):** Continue dancing.
- **Training Method:** HIIT + steady-state cardio, resistance training with increased weights.
- **Intensity:** Moderate to high (65-75% max HR during intervals).
- **Considerations:** Ensure adequate recovery between HIIT sessions, especially if fatigue sets in.

Week 7: Refining Strength and Cardiovascular Capacity

- **Training Frequency:** 5 days/week
- **Duration per Session:** 60 minutes
- **Training Type:**
    - **Aerobics (3 sessions):** Increase to 45 minutes/session, with a focus on maintaining intensity throughout.
    - **Resistance Training (2 sessions):** Incorporate new movements (e.g., deadlifts, rows).
    - **Dancing (1 session):** Continue dancing.
- **Training Method:** Alternating HIIT with moderate intensity.
- **Intensity:** Moderately high (65-75% max HR).
- **Considerations:** Continue to track glucose levels, especially on resistance training days.

Week 8: Peak Intensity

- **Training Frequency:** 5 days/week
- **Duration per Session:** 60 minutes
- **Training Type:**
    - **Aerobics (2 sessions):** HIIT for 30 minutes, followed by steady-state exercise.
    - **Resistance Training (2 sessions):** Focus on strength with higher weights and fewer reps.
    - **Dancing (1 session):** Continue dancing.
- **Training Method:** Combination of strength and cardiovascular work.
- **Intensity:** High (70-80% max HR).
- **Considerations:** Plan carbohydrate snacks pre- and post-workout to prevent hypoglycemia.

Weeks 9–12: Optimization and Long-Term Maintenance

Week 9–12: Maintenance Phase

- **Training Frequency:** 5–6 days/week
- **Duration per Session:** 60 minutes
- **Training Type:**
    - **Aerobics (3 sessions/week):** High-intensity intervals and longer duration aerobic sessions (45–60 minutes).
    - **Resistance Training (2 sessions/week):** Continue to increase weight for strength improvements.
    - **Dancing (1 session/week):** Maintain the dancing routine.
- **Training Method:** Maintain a mix of HIIT and strength training.
- **Intensity:** Moderate to high (70-80% max HR).
- **Considerations:** Focus on maintaining consistency. Keep glucose-monitoring practices and proper hydration in place.

Conclusion:

This 12-week exercise plan is designed to help the individual achieve optimal health and fitness benefits while adhering to ADA and ACSM guidelines. It takes into account the individual's type 2 diabetes, medication, fitness level, and personal preferences. The program progressively increases intensity and introduces various training methods, ensuring both safety and enjoyment in the process. Regular glucose monitoring is advised to prevent hypoglycemia, and the plan offers flexibility in managing the demands of both aerobic and resistance training."

Patient 2, Prompt 3

12-Week Exercise Plan for a 51-Year-Old Female with Type 2 Diabetes, BMI 45, and High Blood Pressure

Overview:
This 12-week exercise plan is designed to improve insulin sensitivity, promote cardiovascular health, and support blood pressure management while focusing on gradual weight reduction. The program follows ADA and ACSM guidelines, ensuring a safe progression for someone with a low fitness level, type 2 diabetes, and hypertension. The plan focuses on fitness training as preferred by the individual and includes a balance of aerobic and resistance exercises. Emphasis is placed on slow progression and consistency, with modifications to account for her medical conditions and medication.

Training Goals for the 12-Week Period

- **Improve cardiovascular health** through gradual aerobic conditioning.
- **Enhance insulin sensitivity** and glycemic control via regular physical activity.
- **Promote weight loss**, aiming for gradual and steady weight reduction.
- **Improve strength and endurance** through fitness and resistance training.
- **Support blood pressure management** by incorporating low- to moderate-intensity activities that are safe for hypertension.

Weeks 1–4: Establishing the Foundation

Week 1: Introduction to Movement

- **Training Frequency:** 3 days/week
- **Duration per Session:** 20–30 minutes
- **Training Type:**
  - **Aerobics (2 sessions):** Low-impact walking or cycling at a slow pace for 20 minutes/session.
  - **Resistance Training (1 session):** Light bodyweight exercises (e.g., chair squats, wall push-ups) for 15 minutes, focusing on mobility and form.
- **Training Method:** Steady-state cardio and light resistance training.
- **Intensity:** Low (40-50% max HR); the focus is on gentle movement.
- **Considerations:** Monitor blood pressure before and after sessions. Keep sessions short to prevent fatigue and maintain motivation.

Week 2: Gradually Increasing Effort

- **Training Frequency:** 3–4 days/week

- **Duration per Session:** 30 minutes
- **Training Type:**
  - **Aerobics (2 sessions):** Walking or cycling at a moderate pace for 25 minutes.
  - **Resistance Training (1 session):** Continue with bodyweight exercises, adding 1-2 more exercises like seated leg extensions or light dumbbell curls.
  - **Stretching (1 session):** 15 minutes of flexibility and stretching exercises to improve mobility.
- **Training Method:** Steady-state aerobic and beginner resistance exercises.
- **Intensity:** Low to moderate (50-55% max HR).
- **Considerations:** Encourage hydration and manage blood glucose, especially with increased activity.

Week 3: Building Consistency

- **Training Frequency:** 4 days/week
- **Duration per Session:** 30–35 minutes
- **Training Type:**
  - **Aerobics (2 sessions):** Walking or stationary cycling, increasing to 30 minutes/session at a comfortable, steady pace.
  - **Resistance Training (2 sessions):** Continue bodyweight and light dumbbell exercises, increasing to 2 sets of 10–12 reps for each movement.
- **Training Method:** Steady-state aerobic exercise and beginner resistance training with slight intensity progression.
- **Intensity:** Moderate (55-60% max HR).
- **Considerations:** Maintain good posture during resistance training and focus on controlled movement.

Week 4: Introducing Variety

- **Training Frequency:** 4 days/week
- **Duration per Session:** 35 minutes
- **Training Type:**
  - **Aerobics (2 sessions):** Low-impact aerobics or brisk walking for 30 minutes.
  - **Resistance Training (2 sessions):** Add resistance bands or light weights (1-2 kg) to resistance exercises, maintaining 2 sets of 10-12 reps.
- **Training Method:** Combination of low-impact aerobic exercises and beginner resistance with additional load.
- **Intensity:** Moderate (55-65% max HR).

- **Considerations:** Monitor breathing and avoid straining during resistance training.

Weeks 5–8: Building Strength and Endurance

Week 5: Expanding Duration

- **Training Frequency:** 4 days/week
- **Duration per Session:** 40 minutes
- **Training Type:**
    - **Aerobics (2 sessions):** Walking, cycling, or light aerobics for 35 minutes/session.
    - **Resistance Training (2 sessions):** Increase resistance, aiming for 2–3 sets of 12–15 reps. Include exercises like shoulder presses, seated rows with resistance bands.
- **Training Method:** Steady-state aerobic work with more challenging resistance exercises.
- **Intensity:** Moderate (55-65% max HR).
- **Considerations:** Pay attention to blood sugar control and monitor fatigue after sessions.

Week 6: Introduction to Interval Training

- **Training Frequency:** 4–5 days/week
- **Duration per Session:** 40 minutes
- **Training Type:**
    - **Aerobics (2 sessions):** Add light intervals to walking or cycling (e.g., 1 minute faster pace, 2 minutes recovery).
    - **Resistance Training (2 sessions):** Continue with light weights or resistance bands, increasing to 3 sets for key movements.
- **Training Method:** Low-intensity interval training (LIIT) and strength training.
- **Intensity:** Moderate (55-65% max HR during intervals).
- **Considerations:** Avoid any sudden changes in intensity due to hypertension. Allow more rest if necessary.

Week 7: Increasing Intensity Gradually

- **Training Frequency:** 5 days/week
- **Duration per Session:** 40–45 minutes
- **Training Type:**

- **Aerobics (3 sessions):** Continue with low-impact intervals, increasing to 35 minutes/session.
- **Resistance Training (2 sessions):** Progress to moderate-intensity strength exercises using 3 sets of 12–15 reps.

- **Training Method:** Alternating aerobic intervals and resistance training.
- **Intensity:** Moderate (60-70% max HR).
- **Considerations:** Pay attention to how the body responds to added intensity. Check blood pressure regularly.

Week 8: Pushing Endurance

- **Training Frequency:** 5 days/week
- **Duration per Session:** 45 minutes
- **Training Type:**
    - **Aerobics (3 sessions):** Walking or cycling at a moderate pace with intervals (e.g., 2 minutes fast, 3 minutes slow).
    - **Resistance Training (2 sessions):** Continue strength training with resistance bands or free weights, increasing resistance if tolerable.
- **Training Method:** Moderate aerobic exercise with longer intervals and strength training.
- **Intensity:** Moderate (60-70% max HR).
- **Considerations:** Be mindful of heart rate during more vigorous aerobic activities.

Weeks 9–12: Refinement and Long-Term Adaptation

Week 9: Balancing Strength and Cardio

- **Training Frequency:** 5 days/week
- **Duration per Session:** 45–50 minutes
- **Training Type:**
    - **Aerobics (3 sessions):** Walking or low-impact aerobics for 40 minutes.
    - **Resistance Training (2 sessions):** Focus on compound movements like squats, rows, and chest presses with moderate weights.
- **Training Method:** Steady-state and interval-based cardio, resistance training with focus on strength.
- **Intensity:** Moderate to high (60-75% max HR).
- **Considerations:** Ensure a good warm-up before strength sessions and proper hydration.

Week 10: Progressing to Greater Challenge

- **Training Frequency:** 5 days/week
- **Duration per Session:** 50 minutes
- **Training Type:**
  - **Aerobics (3 sessions):** Include longer intervals of brisk walking or cycling (3 minutes fast, 3 minutes recovery).
  - **Resistance Training (2 sessions):** Continue with moderate weights, focusing on form and increasing difficulty (3 sets of 12-15 reps).
- **Training Method:** Combination of interval-based cardio and strength workouts.
- **Intensity:** Moderate to high (65-75% max HR).
- **Considerations:** Keep intensity manageable, especially for hypertension and blood sugar control.

Week 11: Refining Strength and Endurance

- **Training Frequency:** 5 days/week
- **Duration per Session:** 50–55 minutes
- **Training Type:**
  - **Aerobics (3 sessions):** Maintain interval training, with a focus on increasing pace or incline on walks.
  - **Resistance Training (2 sessions):** Include more advanced strength exercises like deadlifts (using resistance bands) and overhead presses.
- **Training Method:** Interval training and advanced resistance work.
- **Intensity:** Moderate to high (65-75% max HR).
- **Considerations:** Maintain good breathing techniques and posture during strength exercises.

Week 12: Peak Week for Fitness

- **Training Frequency:** 5–6 days/week
- **Duration per Session:** 55–60 minutes
- **Training Type:**
  - **Aerobics (3 sessions):** Continue interval training with 45-minute sessions.
  - **Resistance Training (2–3 sessions):** Focus on improving strength and endurance with weights or resistance bands.
- **Training Method:** Interval and steady-state cardio combined with strength-focused exercises.
- **Intensity:** Moderate to high (70-75% max HR).

- **Considerations:** Pay close attention to energy levels and recovery time. Track weight and blood pressure progress over the past 12 weeks.

Conclusion:

This 12-week exercise plan is designed to safely and gradually build strength, endurance, and cardiovascular fitness for a female with type 2 diabetes, high BMI, and hypertension. The plan adheres to ADA and ACSM guidelines and considers the individual's low fitness level, medications, and preferences for fitness training. Adjustments can be made based on ongoing health assessments and personal progress throughout the program.

Patient 3, Prompt 3

12-Week Exercise Plan for a 65-Year-Old Male with Type 2 Diabetes Mellitus, Proliferative Retinopathy, and Diabetic Foot Syndrome

---

Overview:

This exercise plan is designed for a 65-year-old male with type 2 diabetes, a BMI of 31, and complications including proliferative retinopathy and diabetic foot syndrome (Wagner 0). The plan focuses on cycling (the individual's preferred exercise), incorporates low-impact cardio, resistance training, and aims to safely improve cardiovascular health, insulin sensitivity, and mobility. Special attention has been given to the individual's low fitness level, medication (metformin and insulin), and secondary complications.

Training Goal:

The goal of this 12-week program is to:

- Improve insulin sensitivity and glucose management
- Enhance cardiovascular health and circulation
- Build muscle strength and endurance to support diabetic foot care
- Promote safe weight management

Precautions & Considerations:

- **Proliferative Retinopathy:** Avoid high-intensity exercises and activities that involve heavy lifting or straining to prevent worsening eye complications.
- **Diabetic Foot Syndrome (Wagner 0):** Monitor foot health regularly. Wear appropriate footwear, and avoid high-impact exercises that could increase the risk of ulcers.
- **Medication Considerations:** Since the individual is on metformin and insulin, it's important to monitor blood sugar levels before, during, and after exercise to prevent hypoglycemia.

- **Progression:** Gradually increase intensity to prevent injury or undue stress on the body.

Plan Structure:

- **Frequency:** 3–4 times per week (increasing gradually).
- **Duration:** Starting at 20 minutes per session, increasing to 45 minutes over 12 weeks.
- **Intensity:** Low to moderate, gradually progressing.

Weeks 1–4: Foundation Phase

Week 1–2: Introduction to Activity

- **Goal:** Build foundational endurance and establish routine.
- **Cycling:** 3 days per week, 20 minutes per session (low intensity, steady-state cardio).
- **Resistance Training:** 1 day per week (using light resistance bands or bodyweight). Focus on major muscle groups (legs, core, arms).
    - Exercises: Seated leg lifts, seated bicep curls, wall push-ups (2 sets of 10–12 reps).
- **Foot Care:** Perform daily foot checks and wear supportive footwear.

Week 3–4: Building Routine

- **Cycling:** 3–4 days per week, 25–30 minutes per session (low to moderate intensity).
- **Resistance Training:** 2 days per week.
    - Exercises: Continue with seated exercises, but increase resistance if comfortable. Add seated calf raises and seated rows with resistance bands.
- **Balance Training:** Add 5–10 minutes of balance exercises such as standing on one leg (hold onto a stable surface for support).

Weeks 5–8: Strength and Endurance Phase

Week 5–6: Increasing Duration

- **Cycling:** 4 days per week, 30–35 minutes per session (moderate intensity, increase cadence slightly).
    - Adjust seat height and bike fit for comfort and safety.
- **Resistance Training:** 2 days per week.
    - Exercises: Continue strengthening exercises. Add seated leg presses (with a light weight if available), standing lateral raises with resistance bands (2–3 sets of 10–12 reps).

- **Core Training:** Introduce 5–10 minutes of core stability exercises such as seated marches and seated pelvic tilts.

Week 7–8: Gradual Progression

- **Cycling:** 4 days per week, 35–40 minutes per session (moderate intensity).
- **Resistance Training:** 2 days per week.
    - Exercises: Increase resistance or reps based on comfort and progress. Focus on functional movements (e.g., sit-to-stand from a chair).
- **Balance and Core:** Increase to 10 minutes per session.

Weeks 9–12: Optimization Phase

Week 9–10: Enhancing Cardiovascular Endurance

- **Cycling:** 4 days per week, 40–45 minutes per session (moderate intensity).
    - Introduce some interval training (e.g., 1 minute of slightly faster pedaling, followed by 3 minutes of easy pedaling).
- **Resistance Training:** 2–3 days per week.
    - Focus on slightly heavier resistance or adding an additional set (3 sets of 10–12 reps).
- **Core and Balance Training:** 10–15 minutes per session.

Week 11–12: Final Push

- **Cycling:** 4 days per week, maintain 45 minutes per session. Continue with moderate intensity, focusing on longer intervals (2 minutes moderate effort, 2 minutes recovery).
- **Resistance Training:** 3 days per week.
    - Continue to increase reps or resistance based on progress. Incorporate more standing exercises if balance has improved.
- **Balance and Core Training:** Maintain 15 minutes per session, incorporating more dynamic balance challenges like heel-to-toe walking.

ADA & ACSM Guidelines Consideration:

- **ADA Guidelines:** The plan includes low- to moderate-intensity aerobic and resistance exercises, which align with ADA's recommendations to improve glucose control and insulin sensitivity. Cycling, a low-impact aerobic exercise, is emphasized to reduce strain on the feet, important for someone with diabetic foot syndrome. Resistance training is included to enhance muscle strength and support overall mobility.
- **ACSM Guidelines:** The plan ensures gradual progression to build cardiovascular health and improve muscle strength, while avoiding high-intensity exercises due to the

individual’s retinopathy. Special care is taken to prevent excessive strain on the eyes and feet.

Safety & Monitoring:

- **Blood Sugar Monitoring:** Check glucose levels before and after exercise to prevent hypoglycemia, especially when insulin is being used.
- **Foot Care:** Perform daily checks, avoid high-impact exercises, and wear proper footwear to prevent ulcers or injury.
- **Eye Care:** Avoid exercises that strain the eyes (e.g., high-resistance strength training, high-impact sports), in line with recommendations for individuals with proliferative retinopathy.

Summary:

This 12-week program balances aerobic (cycling) and resistance exercises, ensuring a gradual increase in intensity and duration. It adheres to safety precautions for managing diabetes, proliferative retinopathy, and diabetic foot syndrome. The plan’s gradual progression, low impact, and emphasis on health monitoring provide a safe and structured path to improved cardiovascular health, insulin sensitivity, and overall fitness.
